# Metabolites of Kimchi Lactic Acid Bacteria, Indole-3-Lactic Acid, Phenyllactic Acid, and Leucic Acid, Inhibit Obesity-Related Inflammation in Human Mesenchymal Stem Cells

**DOI:** 10.4014/jmb.2308.08015

**Published:** 2023-10-31

**Authors:** Moeun Lee, Daun Kim, Ji Yoon Chang

**Affiliations:** 1 Fermentation Regulation Research Group, World Institute of Kimchi, Gwangju 61755, Republic of Korea; 2 Biomodulation Major, Department of Agricultural Biotechnology, Seoul National University, Seoul 08826, Republic of Korea; 3 Department of Food and Nutrition, Chosun University, Gwangju 61452, Republic of Korea; 4 Division of Food Science and Technology, Gyeongsang National University, Jinju 52828, Republic of Korea

**Keywords:** *Companilactobacillus allii* WiKim39, *Lactococcus lactis* WiKim0124, kimchi starter, human mesenchymal stem cells, lipid accumulation

## Abstract

Given the diversity of vegetables utilized in food fermentation and various lactic acid bacteria (LAB) populations in these materials, comprehensive studies on LAB from vegetable foods, including kimchi, are imperative. Therefore, this study aimed to investigate the obesity-related inflammation response of three metabolites—phenyllactic acid (PLA), indole-3-lactic acid (ILA), and leucic acid (LA)—produced by LAB (*Companilactobacillus allii* WiKim39 and *Lactococcus lactis* WiKim0124) isolated from kimchi. Their effects on tumor necrosis factor-α-induced changes in adipokines and inflammatory response in adipose-derived human mesenchymal stem cells were examined. The study results showed that PLA, ILA, and LA, particularly PLA, effectively reduced lipid accumulation and triglyceride, glycerol, free fatty acid, and adiponectin levels. Furthermore, the identified metabolites were found to modulate the expression of signaling proteins involved in adipogenesis and inflammation. Specifically, these metabolites were associated with enriched expression in the chemokine signaling pathway and cytokine-cytokine receptor interaction, which are critical pathways involved in regulating immune responses and inflammation. PLA, ILA, and LA also suppressed the secretion of pro-inflammatory cytokines and several inflammatory markers, with the PLA-treated group exhibiting the lowest levels. These results suggest that PLA, ILA, and LA are potential therapeutic agents for treating obesity and inflammation by regulating adipokine secretion and suppressing pro-inflammatory cytokine production.

## Introduction

Kimchi, a representative fermented food in Korea, is well-known for its rich nutritional content, including vitamins, minerals, dietary fibers, and other biological compounds [1]. Numerous studies have highlighted the potential health benefits of bioactive compounds in kimchi, including their anti-oxidant [2], anti-obesity [3], and anti-cancer effects [4]. These benefits are largely attributed to specific metabolites produced by the lactic acid bacteria (LAB) found in kimchi [5]. Compared to LAB from dairy sources, LAB from plant materials such as kimchi and vegetable products has not been extensively studied. Given the diversity of vegetable materials utilized in food fermentation and various LAB populations in these materials, comprehensive studies on LAB from vegetable foods, including kimchi, are imperative to understand the role of LABs in contributing to the health-promoting properties of kimchi. In addition to the isolation of novel species from diverse ethnic foods, the identification of their metabolites should include studies of the biochemical and functional properties of these metabolites [6].

A previous study revealed that functional LAB (*Companilactobacillus allii* WiKim39 and *Lactococcus lactis* WiKim0124) isolated from kimchi exhibited immunomodulatory functions and improved anti-oxidant properties and anti-obesity effects [7]. Furthermore, 3-phenyllactic (PLA), indole-3-lactic (ILA), and 2-hydroxyisocaproic acids (d-leucic acid) (LA) have been detected in the culture filtrate of LAB (WiKim39 and WiKim0124)-fermented products, including deMan, Rogosa, and Sharpe (MRS) broth, starter kimchi, and fermented vegetable products [8].

PLA is a compound produced from phenylalanine and shares similarities with phenylpyruvate as an intermediate substrate. Its levels are controlled by lactate dehydrogenase, the primary enzyme responsible for converting pyruvate into lactic acid in the glycolytic pathway [9]. Moreover, PLA downregulates pro-inflammatory cytokines, stimulates anti-inflammatory cytokines, and modulates colonic microbiota dysbiosis by increasing beneficial bacteria populations [10]. ILA is a primary metabolite of tryptophan produced by *Bifidobacterium* strains isolated from human infants. It plays a specific role in regulating the immune system and facilitating interactions between the host and the microbiome [11]. However, its role in LAB derived from fermented vegetable products remains unclear. LA, an amino acid-derived metabolite from leucine, has been detected in several fermented foods [12] and has demonstrated efficacy against inflammation and bacterial infections [13]. However, the obesity-related immunomodulatory properties of these metabolites have not been studied.

Inflammation is a complex response of the immune system that can cause damage if not properly regulated. Adipose or fat tissue plays a critical role in energy storage and metabolism [14]. However, excessive adipose tissue accumulation can induce chronic low-grade inflammation, which can contribute to the development of obesity-related complications such as insulin resistance, type 2 diabetes, and cardiovascular diseases [15]. Macrophages in adipose tissue secrete increased levels of pro-inflammatory cytokines, leading to a prolonged, low-level inflammation associated with obesity and resulting in impairments in insulin signaling. Recently, hepatokines and myokines have garnered attention as co-regulators of metabolic diseases [16]. While potential triggers have been proposed, the precise contributors to macrophage accumulation in adipose tissue during obesity remain elusive. The exploration of these inflammatory pathways could unveil promising treatment avenues for metabolic diseases associated with obesity.

This study aimed to explore the underlying mechanisms of obesity-related inflammation, including the impact of pro-inflammatory molecules on metabolic functions. Based on a previous study, it was hypothesized that the enhanced production of functional bioactive compounds, such as PLA, ILA, and LA, may be primarily responsible for the immunomodulatory functions, improved anti-oxidant properties, and anti-obesity effects observed in functional LAB isolated from kimchi [7]. Thus, the effect of these kimchi LAB-derived metabolites on adipogenesis and cellular adipokine profile in mature human adipose-derived mesenchymal stem cells (hMSCs) were investigated, and the potential therapeutic approaches to target obesity-related inflammation and improve metabolic health were discussed.

## Materials and Methods

### Bacterial Culture Condition and Quantification of PLA, ILA, and LA

*C. allii* WiKim39 (GenBank ID: NR_159087.1) and *Lactococcus lactis* WiKim0124 (GenBank ID: MZ424472.1) were previously isolated from kimchi. LAB strains were cryopreserved in MRS broth with 15% glycerol at −80°C and revived in MRS broth at 30°C for 24 h before the experiment. The bacterial supernatants were filtered using a 0.45 μm filter membrane and subjected to ultra-performance liquid chromatography quadrupole time-of-flight tandem mass spectrometry (UPLC-QTOF-MS/MS). The TSQ Altis triple quadrupole mass spectrometer (Thermo Fisher Scientific, USA) was connected to the Vanquish UPLC system (Thermo Fisher Scientific) with a Waters CORTECS T3, C18 column (2.1 × 150 mm, 1.6 μm; Waters, USA). The high-resolution experiment in negative ion mode was conducted using the QTOF-MS/MS through an electrospray ionization (ESI) interface. The Waters Acquity photodiode array (PDA) detector was used as the detection system, which was configured to operate within the 200–400 nm wavelength range. The UPLC separation employed an elution program with water containing 10 mM ammonium acetate as eluent A and methanol as eluent B. The gradient elution program comprised the following steps: 15% B from 0 to 0.4 min and 15% B from 0.5 to 7 min. At 7 to 7.5 min, the gradient changed from 15% to 100% B, followed by 100% to 15% B at 8 min, and equilibration with 15% B for 5 min at a flow rate of 0.2 ml/min. The column temperature was set at 45°C, and the auto-sampler was maintained at 4°C. The detailed experimental conditions are described in a previous study [8]. Thermo Xcalibur software (Thermo Fisher Scientific) was used for data acquisition and analysis. The standard solutions of PLA, ILA, and LA were purchased from Sigma-Aldrich (USA).

### Cell Culture and Adipogenic Differentiation

hMSCs were purchased from the American Type Culture Collection (ATCC) (PCS-500-011) and maintained in culture at 37°C and 5% CO_2_ using the MesenCult proliferation kit (StemCell Technologies, Canada). At passage three, the cells were collected and grown in 6-well plates at 1.5 × 10^4^ cells/ml density using MesenCult medium containing the adipogenic differentiation kit (StemCell Technologies) to differentiate into adipocytes. The cells were treated with the standard substances — LA, ILA, and PLA —at concentrations ranging from 0.31 to 10 mM, and the culture medium was refreshed every 2 days until the hMSCs were matured. Subsequently, on day 21, the matured hMSCs were pretreated with PLA, ILA, and LA for 6 h before treatment with tumor necrosis factor-α (TNF-α) (10 ng/ml). The conditioned medium was collected after 24 h. To assess lipid accumulation level, cells were fixed in 10% formaldehyde in phosphate-buffered saline (PBS) and washed twice. Then, they were stained with Oil Red O (ORO) solution (Sigma-Aldrich) for 10 min and repeatedly rinsed with distilled water. Images of cells were captured at a 25× magnification using a digital camera attached to an inverse phase contrast microscope (Leica TP 1020, Germany).

### Measurement of Triglyceride, Glycerol, and Free Fatty Acid Content

Cellular triglyceride (TG), glycerol, and free fatty acid (FFA) content were determined using commercially available assay kits (Abcam, China). For the TG assay, cells were lysed in a lysis buffer containing 1% Triton X-100 in PBS, and a TG colorimetric assay kit was used. Similarly, for the glycerol and FFA assay, a commercial glycerol and FFA analysis kit was used to measure glycerol and FFA released from the cells into the medium. The amounts of TG, glycerol, and FFA were normalized to the cellular protein content determined using the bicinchoninic acid (BCA) protein assay kit (Thermo Scientific).

### Secreted Proteomic Profile Using Cytokine and Adipokine Antibody Arrays

Fully matured hMSCs were harvested, and their lysates were prepared following the protocol described in a previous study [7]. Protein concentration was determined using the BCA assay. The human obesity antibody membrane array and cytokine membrane arrays (Abcam, UK) were used to detect multiple proteins following the manufacturer's protocol. Briefly, the array membranes were first treated with the provided blocking buffer for 30 min. Then, 1 ml of cell supernatant per array membrane was added, followed by incubation with a biotinylated detection antibody. After washing, the membranes were incubated with horseradish peroxidase-conjugated streptavidin (diluted 1:100) on a rocker. Finally, the spots on the array membranes were scanned and quantified using densitometry using a Chemidoc imaging system (Bio-Rad, USA).

### Bioinformatic Analysis

Proteins that exhibited a log_2_ fold change of ≥ |1| between the adipogenic differentiation group (CON) and the pre-adipocytic group (NOR) were considered differentially expressed proteins (DEPs). Specific information on all proteins identified in this study was obtained from the UniProt database (https://www.uniprot.org/). DAVID tools (https://david.ncifcrf.gov/) were used to perform the Kyoto Encyclopedia of Genes and Genomes (KEGG) analysis. Fisher's exact test was performed to determine the significance of KEGG enrichment for multiple testing, and the resulting *p*-value was adjusted using the false discovery rate (FDR) with a cutoff of < 0.05. The most highly enriched DEPs were analyzed using hierarchical clustering and represented on a heatmap using the "pheatmap" package in R (v3.3.2; https://www.r-project.org).

### Cytokine Concentration

To determine cytokine and chemokine levels (interleukin-2 [IL-2], IL-4, IL-6, interferon-γ [IFN-γ], TNF-α, and granulocyte-macrophage colony-stimulating factor [GM-CSF]), the medium from cell culture was collected and analyzed using the Bio-Plex Pro human cytokine 8-plex assay kit (Bio-Rad) following the manufacturer's instructions. This assay was performed in triplicate.

### Quantitative Real-Time Polymerase Chain Reaction

The RNeasy kit (Qiagen, USA) was used to extract RNA, and approximately 10 ng RNA was amplified for quantitative real-time polymerase chain reaction (qRT-PCR) using a QuantiFast SYBR Green RT-PCR Kit (Qiagen). The RNA was reverse-transcribed at 50°C for 10 min and subjected to an activation step at 95°C for 5 min. The amplification process included denaturation at 95°C for 10 s, followed by annealing and extension at 60°C for 30 s for 35 cycles. The expression levels were normalized to those of the actin beta gene. The primers used for qRT-PCR are listed in Table 1.

### Statistical Analysis

The "agricolae" package in R was used to perform Tukey's honest significant difference (HSD) test to analyze statistical significance for group comparisons. Group comparisons were performed using Student's *t*-test in PRISM 6.0 software from GraphPad (USA), and statistically significant results were defined as *p* < 0.05. All experiments were performed in triplicate, and the data were presented as mean ± standard deviation (SD).

## Results

### Production of PLA, ILA, and LA by WiKim39 and WiKim0124

**Fig. 1:**
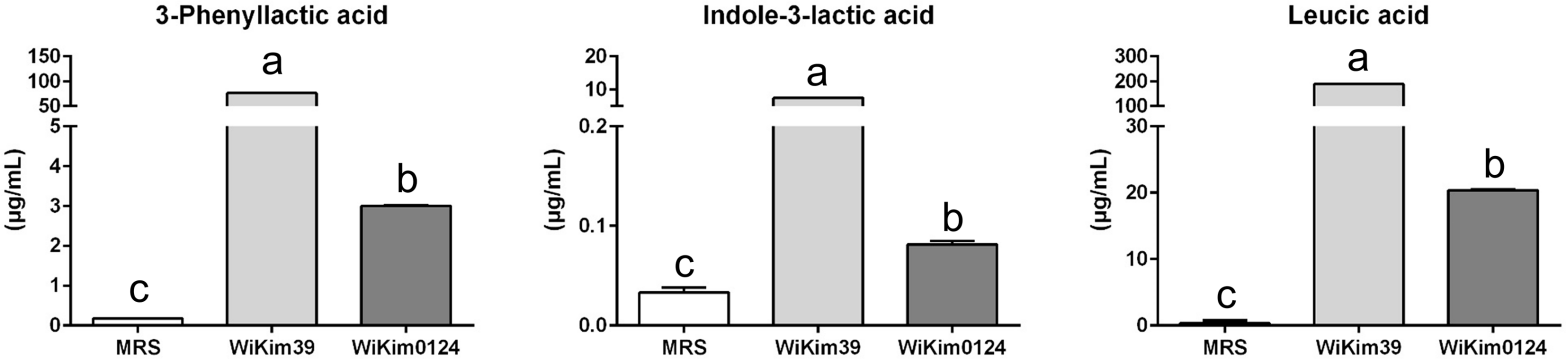
Quantification of PLA, ILA, and LA produced by WiKim39 and WiKim0124 using UPLC-QTOF-MS/MS. UPLC-QTOF-MS/MS, ultra-performance liquid chromatography with quadrupole time-of-flight tandem mass spectrometry.

The target compounds were monitored and quantified using UPLC-QTOF-MS/MS. WiKim39 and WiKim0124 culture supernatants showed significantly higher concentrations of all three compounds than the MRS broth. WiKim39 culture supernatants had the highest concentrations of LA, PLA, and ILA (188.12, 76.22, and 7.48 ng/ml, respectively), whereas the MRS broth had the lowest concentrations at 0.86, 0.18, and 0.04 ng/ml, respectively. WiKim0124 culture supernatants showed the second-highest concentrations of LA, PLA, and ILA (20.78, 3.00, and 0.08 ng/ml, respectively; Fig. 1).

### Effect of PLA, ILA, and LA on Differentiated hMSCs

**Fig. 2:**
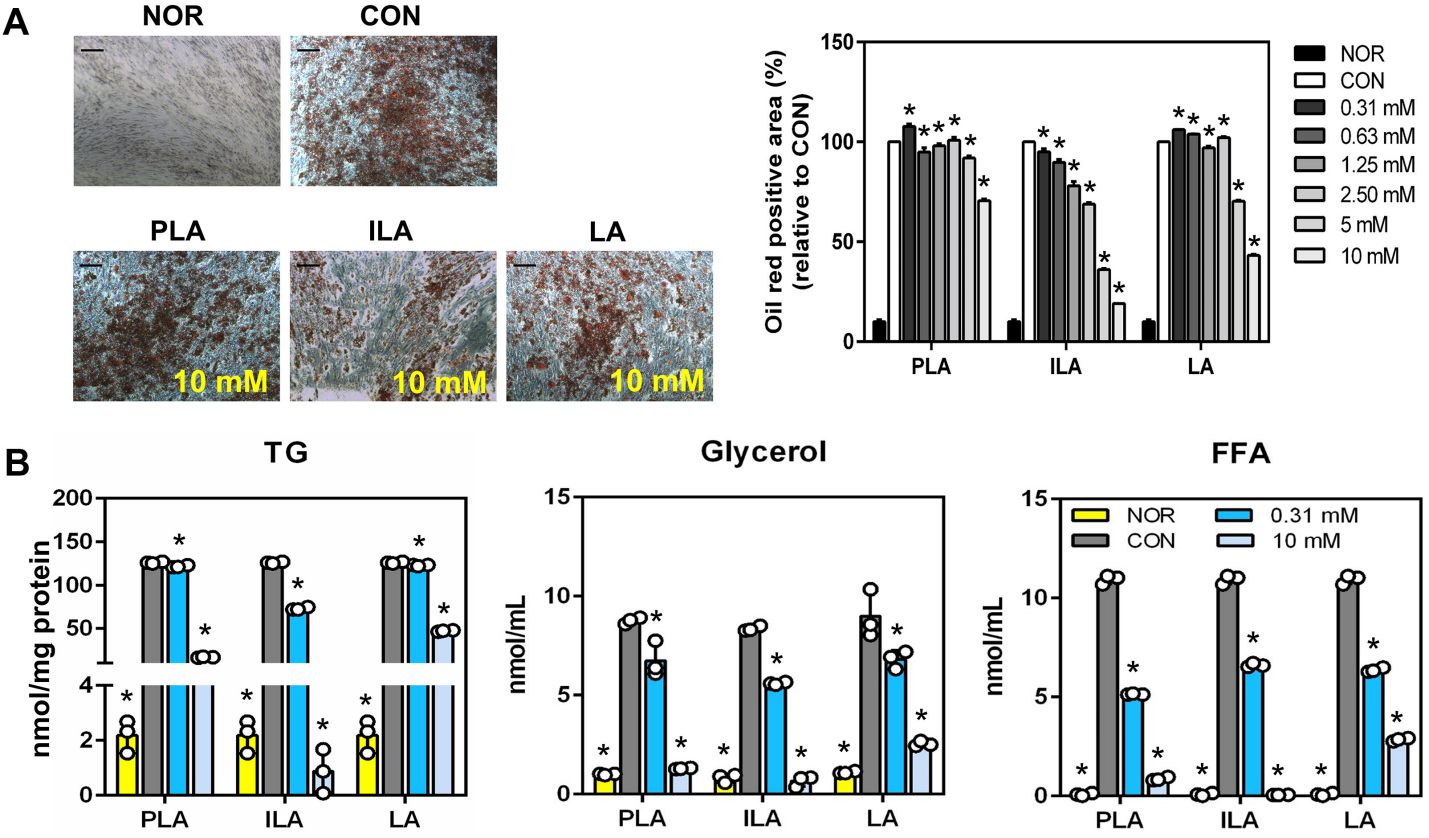
Effect of PLA, ILA, and LA on hMSC differentiation. (**A**) Microscopic pictures. Images were captured using an inverse phase contrast microscope at a magnification of 25×. Scale bars represent 200 μm. (**B**) Triglyceride (TG), glycerol, and free fatty acid (FFA) levels. NOR, normal groups, preadipocytes; CON, control groups, cells were cultured with differentiation medium; compound-treated groups (PLA, ILA, and LA), cells were cultured with differentiation medium and treated with 0.31 to 10 mM concentration with each compound. Data represent the means ± standard deviation (SD). *p < 0.05 indicates statistical differences between CON and experimental groups.

In a previous study, treatment with 10 mM PLA, ILA, and LA did not cause cytotoxicity (data not shown). In this study, an ORO staining assay was performed to visualize lipid accumulation and evaluate the potential anti-adipogenic effect of these compounds (Fig. 2A). Cells in adipogenesis medium for 4 weeks exhibited clusters of cells containing lipid droplets, which were positively stained with ORO. Sample treatment suppressed lipid accumulation in hMSCs, with the highest effect exerted at a concentration of 10 mM ILA. The 10 mM PLA and LA treatment groups exhibited similar patterns. These results indicate that the highest suppression of TG accumulation was in the 10 mM ILA treatment group, followed by 10 mM PLA and LA. A similar pattern was also observed in the glycerol and FFA content (Fig. 2B).

### Identification of Differentially Secreted Cytokines and Adipokines from PLA, ILA, and LA Pretreated Adipocytes

**Fig. 3:**
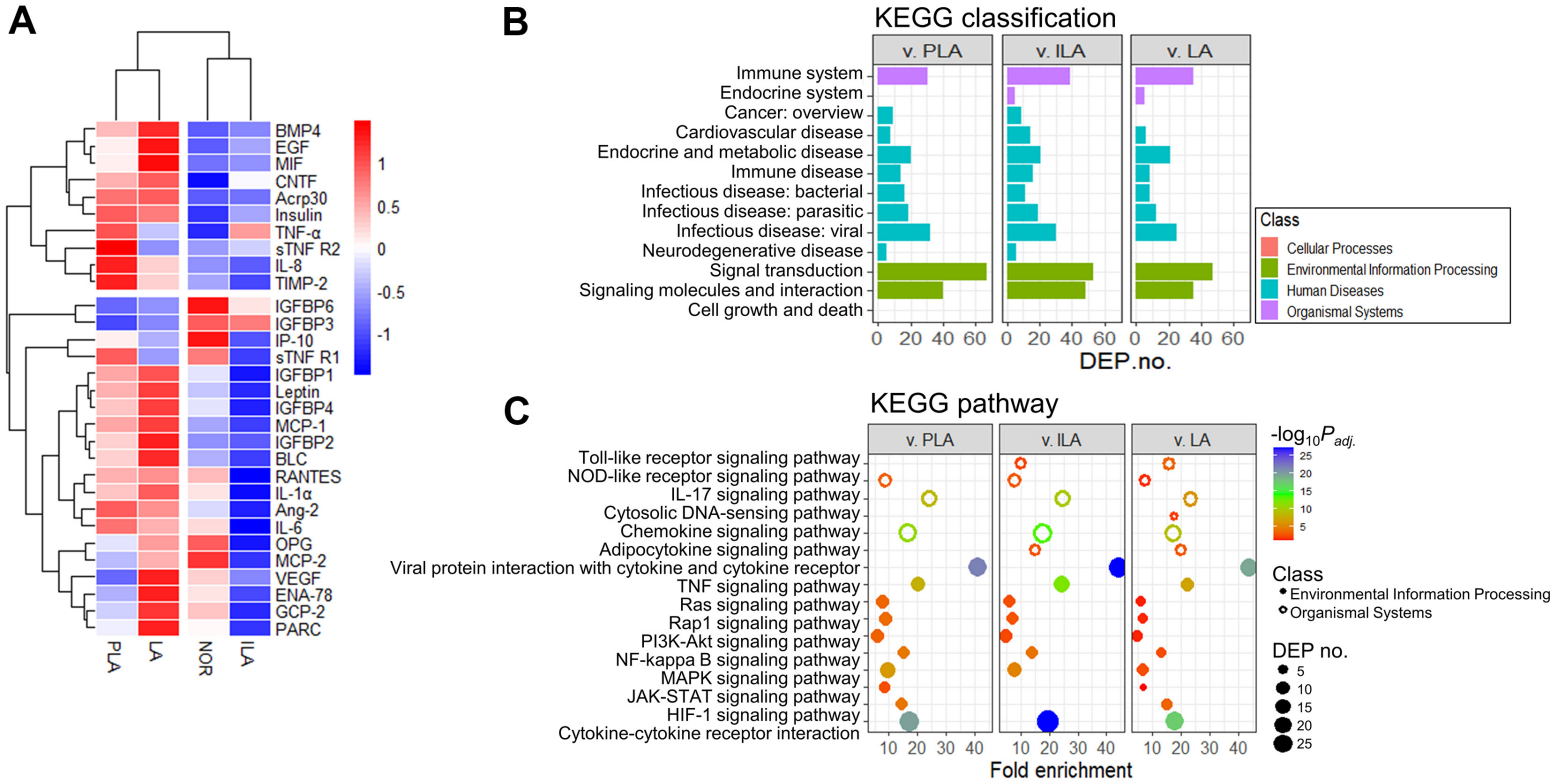
Statistical analysis of antibody array. (**A**) Heat map and hierarchical cluster analysis showing log_2_ fold change of differentially expressed proteins (DEPs) from the control (CON) vs. experimental groups. Thirty proteins (log_2_ fold change) are visualized on the heat map. Different colors represent the different relative abundances of proteins: red and blue represent a higher and lower intensity, respectively. (**B**) Kyoto Encyclopedia of Genes and Genomes (KEGG) classifications. KEGG classification of DEPs from the control (CON) vs. experimental groups. (**C**) Scatter plots showing enriched KEGG pathways in the classes of environmental information processing and organismal systems, comparing pathway enrichment between CON and experimental groups.

Antibody array analysis in TNF-α-induced mature hMSCs supernatant was performed to compare cytokines and adipokines profiles that play a key role in the proliferation and differentiation of preadipocytes. Based on immunoblotting results (Figs. S1 and S2), 51 DEPs with log_2_ fold changes ≥ |1| between the differentiated hMSCs and experimental groups were selected. Fig. 3A shows a heat map and cluster analysis of log_2_ fold changes of the DEPs relative to CON. The DEPs were clustered according to the experimental group and exhibited distinct expression patterns. Most proteins in the experimental groups showed higher expression than those in the NOR and lower expression than those in the CON. The ILA treatment group exhibited a downregulation pattern similar to that of NOR. Bioinformatic analysis was used to clarify the categories in which the DEPs were concentrated. A comparison was made between the DEPs from the CON and sample treatment groups (Fig. 3B and 3C) to conduct the KEGG enrichment analysis. The KEGG enrichment analysis focused on organismal systems and environmental information processing. In the PLA, ILA, and LA treatment groups, the DEPs were linked to immune system activation, signal transduction, and signaling molecules and interactions, including pathways such as cytokine and chemokine signaling. However, only ILA and LA treatment groups demonstrated significant activation of the adipokine signaling pathway.

### Effects of Pretreatment with PLA, ILA, and LA on Inflammatory Cytokines and Gene Expression

**Fig. 4:**
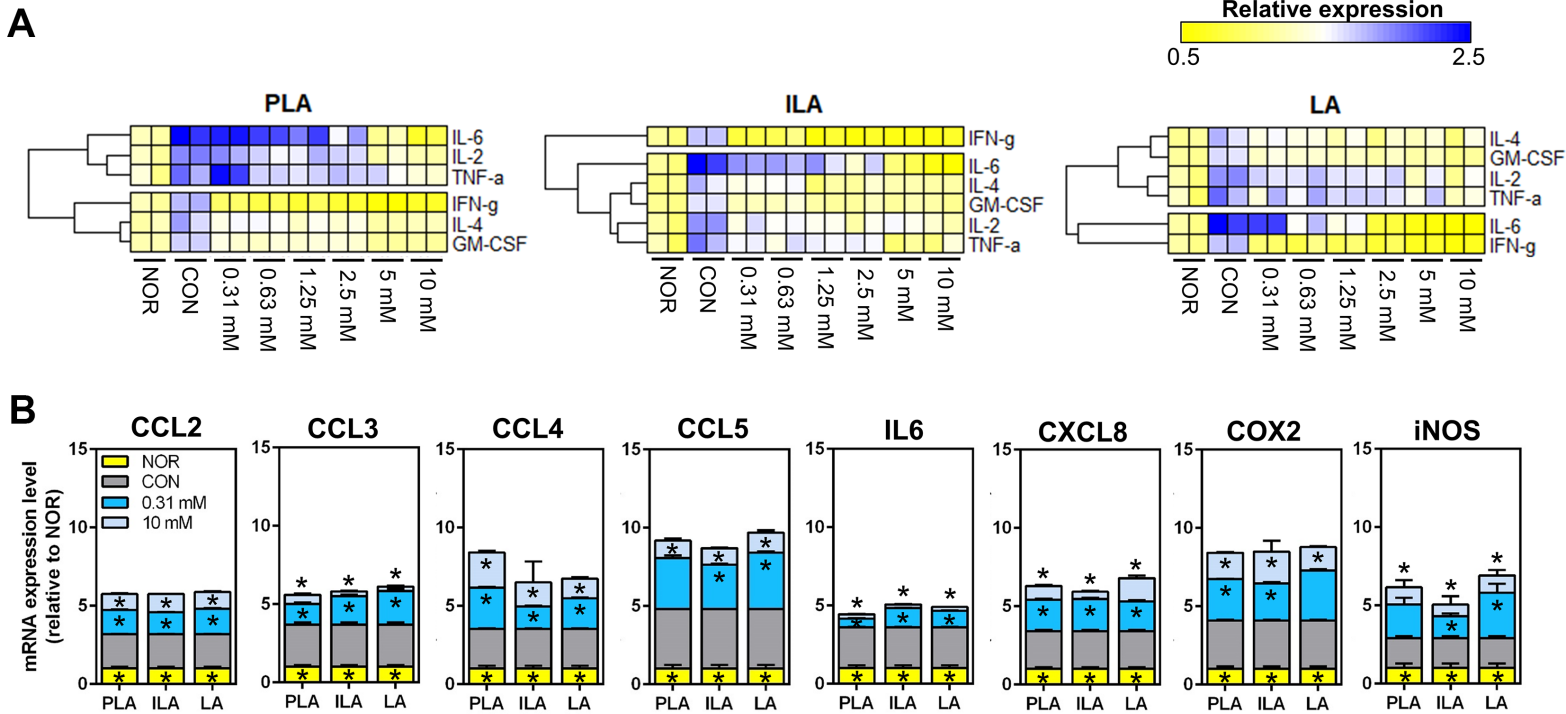
Effects of PLA, ILA, and LA on obesity-related cytokine secretion and gene expressions. (**A**) Heatmap hierarchical clustering analysis of enzyme-linked immunosorbent assay (ELISA) data. NOR, normal groups, preadipocytes; CON, control groups, cells were cultured with differentiation medium; compound-treated groups (PLA, ILA, and LA), cells were cultured with differentiation medium and treated with 0.31 to 10 mM concentration with each compound. (**B**) mRNA expression level. Chemokine (C-C motif) ligand 2 (CCL2), CCL3, CCL4, CCL5, interleukin-6 (IL6), chemokine (C-X-C motif) ligand 8 (CXCL8), cyclooxygenase-2 (COX2), and nitric oxide synthase (iNOS) levels were measured using quantitative realtime polymerase chain reaction (qRT-PCR). Data represent the means ± SD. *p < 0.05 indicates statistical differences between CON and experimental groups.

To investigate the mechanism of obesity-related inflammation, the levels of pro-inflammatory cytokines secreted by differentiated hMSCs and the expression of transcription factors and related genes involved in pro-inflammatory responses were measured using qRT-PCR. Consistent with the antibody array results, PLA, ILA, and LA pretreatment significantly reduced IL-6, IL-4, IL-2, GM-CSF, IFN-γ, and TNF-α (Fig. 4A). The heat map analysis of these enzyme-linked immunosorbent assay (ELISA) data showed different reactivity patterns corresponding to the different sample treatments. Additionally, the expression of the related genes, including chemokine (C-C motif) ligand 2 (CCL2), CCL3, CCL4, CCL5, IL-6, chemokine (C-X-C motif) ligand 8 (CXCL8), cyclooxygenase-2 (COX2), and inducible nitric oxide synthase (iNOS), were decreased in all sample pretreatment groups (Fig. 4B). Despite its low concentration at 0.31 mM, ILA exerted significant effectiveness.

## Discussion

The relationship between adipose tissue, metabolism, and the immune system is evidenced in vitro and in vivo, as they are in proximity to each other within the adipocytes [17]. Cellular components, including lipoteichoic acid, exopolysaccharide, and surface layer proteins, are potential bioactive compounds that could influence the biological function of LAB [18]. A previous study demonstrated that the metabolites (PLA, ILA, and LA) of kimchi-isolated WiKim39 and WiKim0124 LABs are significantly linked to the effectiveness of kimchi in reducing obesity [7]. In this study, the characteristics of obesity-related inflammation in TNF-α-induced differentiated hMSCs were investigated to understand the mechanisms involved in probiotic LAB, WiKim39 and WiKim0124, and determine the contribution of metabolites to the obesity-related inflammatory response.

The study findings suggested that obesity is associated with significant production in microbial-derived PLA, ILA, and LA, particularly ILA. These compounds showed lipolytic activity by effectively reducing intracellular lipid accumulation, including TG, FFA, and glycerol, and modulating inflammation. The antibody array analysis results revealed that most DEPs, under PLA, ILA, and LA treatment, were related to the immune system, signal transduction, and signaling molecules and interactions according to the KEGG classification. Further, KEGG enrichment analysis showed that the DEPs were mainly enriched in pro-inflammatory cytokines, the chemokine signaling pathway, and cytokine-cytokine receptor interaction. However, the adipokine signaling pathway was enriched only in the ILA and LA treatment groups. Adipose tissue serves as an endocrine organ by releasing adipokines, which can be pro-inflammatory or anti-inflammatory. In obesity, the disruption in adipokine secretion induces chronic inflammation and impaired immune response to infections [19]. Thus, the imbalance in adipokine production can also contribute to inflammation in various diseases. PLA may have a weaker effect on adipokine levels than ILA or LA. In line with these findings, the agglomerative hierarchical clustering of the ELISA data revealed similar reactivity patterns, which were reflected in the different main clusters identified in the heat map analysis for each sample treatment. IL-6, IL-2, and TNF-α protein levels were less downregulated in the PLA treatment group than in the ILA and LA treatment groups. A previous study found that 0.1 mM PLA increases the expression of peroxisome proliferators-activated receptor γ2 (PPAR-γ2), CCAAT/enhancer binding protein α (C/EBPα), adiponectin, and sterol regulatory element-binding protein 1 (SREBP-1) genes in 3T3-L1 adipocytes [20], suggesting that low PLA concentrations could promote adipogenesis and regulate fatty acid metabolism through adipogenic gene with specific adipokine activation. An in vitro study showed that ILA, which was produced by *Bifidobacterium longum* subsp. *Infantis* grown on human milk can suppress the secretion of inflammatory cytokines in intestinal epithelial cells [21]. The anti-inflammatory molecule, ILA, has been identified in *B. infantis* and other bacteria, such as *Lactobacillus casei* and *Lactobacillus helveticus*. Studies have shown that ILA has anti-inflammatory activity and can protect keratinocytes against UV-B-induced damage [22, 23]. Similarly, this study demonstrated the strong potential of ILA for inhibiting lipid accumulation in vitro by producing anti-inflammatory molecules. Treatment of macrophage cell lines with 0.1–10 mM ILA produced by *B. infantis* in the presence of TNF-α exhibited cytokine modulatory effects consistent with prior studies [24]. Moreover, the immunomodulatory effects of LA have been observed in a murine model, where it regulates the expression of matrix metalloproteinase 9 (, myeloperoxidase, and developmental endothelial locus 1 [25]; nevertheless, only a limited number of studies have examined its functionalities. In this study, LA significantly improved obesity and related parameters in hMSCs, with efficacy comparable to that of ILA. Based on the antibody array, ELISA, and qPCR results, the reduction in mRNA and protein expression in adipocytes was associated with TNF-α-induced lipid accumulation and inflammation. Nitric oxide (NO) and prostaglandin E2 (PGE2) are important mediators of inflammation. NO is produced by iNOS and can affect various aspects of the inflammatory cascade, whereas PGE2, synthesized by COX2, is considered a strong inflammatory mediator [26]. Chemokines play a significant role in fibrosis, inflammation, and angiogenesis in various pathologies of different organs [27]. TNF-α-induced adipocytes show increased expression of COX2, iNOS, and chemokine genes (CCL2/monocyte chemoattractant protein-1, CCL3/macrophage inflammatory protein-1α [MIP-1α], CCL4/MIP-1β, CCL5/ regulated upon activation, normal T cell expressed and secreted [RANTES], CXCL8/IL-8), which play a key role in the recruitment of monocytes and macrophages [28]. However, significant downregulation of these pathways was observed in all sample treatment groups. Chemokines are a family of small cytokines that serve as signaling proteins to direct cell migration. They play a crucial role in homeostatic and inflammatory responses, with the latter being a protective response to localize and eliminate harmful substances and tissue components [29]. Overall, the results of this study suggest that the regulation of chemokines by PLA, ILA, and LA may positively impact obesity-related inflammation.

In conclusion, this study demonstrated that three metabolites, PLA, ILA, and LA, highly produced by WiKim39 and WiKim0124, can ameliorate obesity and TNF-α-induced inflammation in hMSCs. qPCR and ELISA results revealed that these metabolites reduced lipid accumulation and intracellular TG levels and suppressed inflammation-related transcriptional biomarkers. Furthermore, ILA exhibited greater efficacy than PLA and LA in ameliorating obesity and inflammation. Overall, these findings suggest that WiKim39 and WiKim0124, and their metabolites, are potential functional probiotics and starters for managing obesity and immune function.

## Supplemental Materials

Supplementary data for this paper are available on-line only at http://jmb.or.kr.



## Figures and Tables

**Table 1:** Primer sets used for quantitative real-time PCR in this study.

Target	Forward primer (5'→3')	Reverse primer (5'→3')	Reference
COX2	GTGCAACACTTGAGTGGCTAT	AGCAATTTGCCTGGTGAATGAT	[30]
CCL2	CTTCTGTGCCTGCTGCTCATA	CTTTGGGACACTTGCTGCTG	[31]
CCL3	AGTTCTCTGCATCACTTGCTG	CGGCTTCGCTTGGTTAGGAA	[32]
CCL4	GTCTGTGCTGATCCCAGTGAA	CAGTGACAGTGGACCATCCC	NM_002984.4^a^
CCL5	GGATCAAGACAGCACGTGGA	TCGGGTGACAAAGACGACTG	NM_002985.3
IL-6	ACTCACCTCTTCAGAACGAATTG	CCATCTTTGGAAGGTTCAGGTTG	
CXCL8	TTTTGCCAAGGAGTGCTAAAGA	AACCCTCTGCACCCAGTTTTC	[33]
iNOS	CTCGCTCTGGAAAGACCAGG	GGGACAGGACGTAGTTCAGC	NM_000625.4

^a^From the sequence of the NCBI reference sequence (GenBank Accession No.).
