# Supplementary material for: Metabolites of Kimchi Lactic Acid Bacteria, Indole-3-Lactic Acid, Phenyllactic Acid, and Leucic Acid, Inhibit Obesity-Related Inflammation in Human Mesenchymal Stem Cells
Source: J Microbiol Biotechnol. 2023 Oct 31;34(2):306–13. doi: 10.4014/jmb.2308.08015 (PMC10940772; doi:10.4014/jmb.2308.08015)
Supplement: Supplementary file 1 [file jmb-34-2-306-supple.pdf]

J. Microbiol. Biotechnol.
https://doi.org/10.4014/jmb.2308.08015

Supplementary Figures

(A)

	A	B	C	D	E	F	G	H	I	J	K	L	M	N
1	**POS**	**POS**	**NEG**	**NEG**	4-1BB	ACE-2	Acrp30	Adipsin	AgRP	Ang-2	Angiopoietin-1	ANGPTL4	CRP	ENA-78
2	**POS**	**POS**	**NEG**	**NEG**	4-1BB	ACE-2	Acrp30	Adipsin	AgRP	Ang-2	Angiopoietin-1	ANGPTL4	CRP	ENA-78
3	Fas	FGF-6	Growth Hormone	HCC-4	IFN-γ	IGFBP-1	IGFBP-2	IGFBP-3	IGF-I	IGF-I SR	IL-1 R4/ST2	IL-1 sRI	IL-10	IL-11
4	Fas	FGF-6	Growth Hormone	HCC-4	IFN-γ	IGFBP-1	IGFBP-2	IGFBP-3	IGF-I	IGF-I SR	IL-1 R4/ST2	IL-1 sRI	IL-10	IL-11
5	IL-12	IL-1α	IL-1β	IL-6	IL-6 sR	IL-8	Insulin	IP-10	Leptin R	Leptin	LIF	Lymphotactin	MCP-1	MCP-3
6	IL-12	IL-1α	IL-1β	IL-6	IL-6 sR	IL-8	Insulin	IP-10	Leptin R	Leptin	LIF	Lymphotactin	MCP-1	MCP-3
7	MCSF	MIF	MIP-1β	MSP α	OPG	OSM	PAI-I	PARC	PDGF-AA	PDGF-AB	PDGF-BB	RANTES	Resistin	Serum Amyloid A
8	MCSF	MIF	MIP-1β	MSP α	OPG	OSM	PAI-I	PARC	PDGF-AA	PDGF-AB	PDGF-BB	RANTES	Resistin	Serum Amyloid A
9	SDF-1	sTNF RII	sTNT RI	TECK	TGF-β	TIMP-1	TIMP-2	TNF-α	VEGF	XEDAR	**BLANK**	**BLANK**	**BLANK**	**POS**
10	SDF-1	sTNF RII	sTNT RI	TECK	TGF-β	TIMP-1	TIMP-2	TNF-α	VEGF	XEDAR	**BLANK**	**BLANK**	**BLANK**	**POS**

POS: Positive control, NEG: Negative control, BLANK: No antibody

(B)

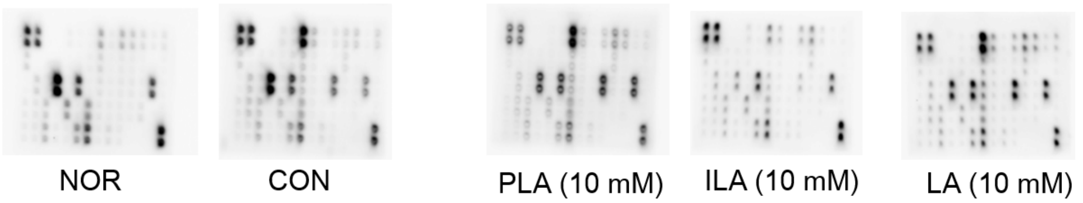

Fig. S1. Human obesity antibody array identification of the effect of PLA, ILA, and LA in cells. (A) Array map used in the panel, **(B)** human antibody array panel used in this study. The signal intensity of the arrays in the panel was analyzed using a densitometer. The relative fold change of individual proteins was calculated after normalization to the positive control of each array (n = 2).

(A)

	A	B	C	D	E	F	G	H	I	J	K	L	M	N
1	**POS**	**POS**	**NEG**	**NEG**	**BLANK**	ANG	BDNF	BLC	BMP4	BMP6	CCL23	CNTF	EGF	Eotaxin1
2	**POS**	**POS**	**NEG**	**NEG**	**BLANK**	ANG	BDNF	BLC	BMP4	BMP6	CCL23	CNTF	EGF	Eotaxin1
3	Eotaxin2	Eotaxin3	FGF-6	FGF-7	Flt-3 Ligand	Fractal-kine	GCP-2	GDN F	GM-CSF	I-309	IFN-γ	IGFBP 1	IGFBP 2	IGFBP4
4	Eotaxin2	Eotaxin3	FGF-6	FGF-7	Flt-3 Ligand	Fractal-kine	GCP-2	GDN F	GM-CSF	I-309	IFN-γ	IGFBP 1	IGFBP 2	IGFBP
5	IGF-1	IL-10	IL-13	IL-15	IL-16	IL-1α	IL-1β	IL-1ra	IL-2	IL-3	IL-4	IL-5	IL-6	IL-7
6	IGF-1	IL-10	IL-13	IL-15	IL-16	IL-1α	IL-1β	IL-1ra	IL-2	IL-3	IL-4	IL-5	IL-6	IL-7
7	Leptin	LIGHT	MCP-1	MCP-2	MCP-3	MCP-4	M-CSF	MDC	MIG	MIP-1δ	MIP-3α	NAP-2	NT-3	PARC
8	Leptin	LIGHT	MCP-1	MCP-2	MCP-3	MCP-4	M-CSF	MDC	MIG	MIP-1δ	MIP-3α	NAP-2	NT-3	PARC
9	PDGF BB	RANTES	SCF	SDF-1α	TARC	TGFβ1	TGFβ3	TNFα	TNFβ	**BLANK**	**BLANK**	**BLANK**	**BLANK**	**POS**
10	PDGF BB	RANTES	SCF	SDF-1α	TARC	TGFβ1	TGFβ1	TNFα	TNFβ	**BLANK**	**BLANK**	**BLANK**	**BLANK**	**POS**

	A	B	C	D	E	F	G	H	I	J	K	L	M	N
1	**POS**	**POS**	**NEG**	**NEG**	**BLANK**	Acrp30	AgRP	ANGPT2	AREG	Axl	bFGF	B-NGF	BTC	CCL28
2	**POS**	**POS**	**NEG**	**NEG**	**BLANK**	Acrp30	AgRP	ANGPT2	AREG	Axl	bFGF	B-NGF	BTC	CCL28
3	CTACK	Dtk	EGFR	ENA-78	Fas	FGF-4	FGF-9	G-CSF	GITR Ligand	GITR	GRO	GROα	HCC-4	HGF
4	CTACK	Dtk	EGFR	ENA-78	Fas	FGF-4	FGF-9	G-CSF	GITR Ligand	GITR	GRO	GROα	HCC-4	HGF
5	ICAM-1	ICAM-3	IGFBP 3	IGFBP 6	IGF-1 sR	IL-1R4	IL-1R1	IL-11	IL-12 p40	IL-12 p70	IL-17	IL-2Rα	IL-6R	IL-8
6	ICAM-1	ICAM-3	IGFBP 3	IGFBP 6	IGF-1 sR	IL-1R4	IL-1R1	IL-11	IL-12 p40	IL-12 p70	IL-17	IL-2Rα	IL-6R	IL-8
7	I-TAC	XCL1	MIF	MIP-1α	MIP-1β	MIP-3β	MSPα	NT-4	OPG	OSM	PLGF	sgp130	sTNFRII	sTNFRI
8	I-TAC	XCL1	MIF	MIP-1α	MIP-1β	MIP-3β	MSPα	NT-4	OPG	OSM	PLGF	sgp130	sTNFRII	sTNFRI
9	TECK	TIMP-1	TIMP-2	THPO	TRAIL R3	TRAIL R4	uPAR	VEGP	VEGP -D	**BLANK**	**BLANK**	**BLANK**	**BLANK**	**POS**
10	TECK	TIMP-1	TIMP-2	THPO	TRAIL R3	TRAIL R4	uPAR	VEGP	VEGP -D	**BLANK**	**BLANK**	**BLANK**	**BLANK**	**POS**

POS: Positive control, NEG: Negative control, BLANK: No antibody

(B)

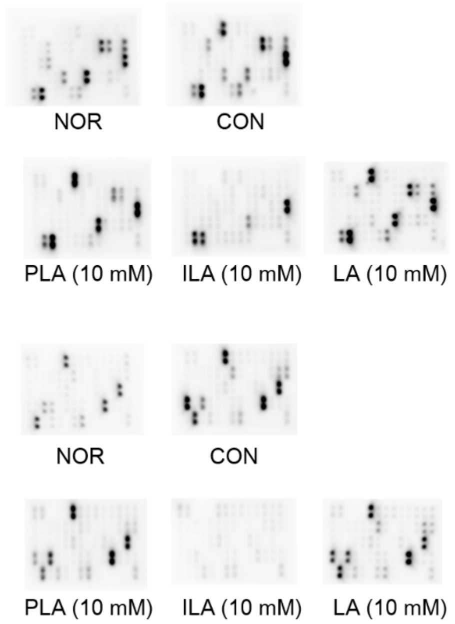

Fig. S2. Human cytokine antibody array identification of the effect of PLA, ILA, and LA in cells. (A) Array map used in the panel, **(B)** human antibody array panel used in this study. The signal intensity of the arrays in the panel was analyzed using a densitometer. The relative fold change of individual proteins was calculated after normalization to the positive control of each array (n = 2).
